# Mast cells and factor XIIIa+ dendrocytes in actinic cheilitis and lip squamous cell carcinoma

**DOI:** 10.1590/1807-3107bor-2024.vol38.0113

**Published:** 2024-12-09

**Authors:** Isadora Luana Flores, José Alcides Almeida de Arruda, Thamiris de Castro Abrantes, Thiago de Oliveira Gamba, Aline Correa Abrahão, Ana Lia Anbinder, Jaqueline Lemes Ribeiro, Ana Carolina Uchoa Vasconcelos, Bruno Augusto Benevenuto de Andrade, Maria Cassia Ferreira de Aguiar, Ana Paula Neutzling Gomes, Lucas Guimarães Abreu, Ricardo Alves Mesquita

**Affiliations:** (a) Universidade Federal do Rio Grande do Sul – UFRGS, School of Dentistry, Department of Oral Pathology, Porto Alegre, RS, Brazil.; (b) Universidade Federal do Rio de Janeiro - UFRJ, School of Dentistry, Department of Oral Diagnosis and Pathology, Rio de Janeiro, RJ, Brazil.; (c) Universidade Federal do Rio Grande do Sul – UFRGS, School of Dentistry, Department of Oral Radiology, Porto Alegre, RS, Brazil.; (d) Universidade Estadual Paulista - Unesp, Institute of Science and Technology, Department of Biosciences and Oral Diagnosis, São José dos Campos, SP, Brazil.; (e) Universidade Federal de Pelotas - UFPel, School of Dentistry, Diagnostic Center for Oral Diseases, Pelotas, RS, Brazil.; (f) Universidade Federal de Minas Gerais - UFMG, School of Dentistry, Department of Oral Surgery, Pathology and Clinical Dentistry, Belo Horizonte, MG, Brazil.; (g) Universidade Federal de Minas Gerais – UFMG, School of Dentistry, Department of Child and Adolescent Oral Health, Belo Horizonte, MG, Brazil.

**Keywords:** Cell Degranulation, Factor XIIIa, Mast Cells, Carcinoma, Squamous Cell

## Abstract

There is an interaction between dendrocytes and mast cells in the skin. However, in elastosis-related diseases such as actinic cheilitis (AC) and lower lip squamous cell carcinoma (LLSCC), this interaction remains unknown. We investigated the presence of intact and degranulated mast cells in AC and LLSCC. Associations of mast cells with factor XIIIa+ dendrocytes and inflammatory infiltrate were assessed. Forty cases of AC (20 with low-grade and 20 with high-grade epithelial dysplasia), 50 cases of LLSCC, and 10 cases of normal oral mucosa were evaluated. Toluidine blue staining was performed to identify mast cells, and mast cell densities were calculated in the inflammatory infiltrate. Factor XIIIa+ dendrocytes were immunohistochemically quantified. The highest ratio of intact/degranulated mast cells density was detected in LLSCC (5.9 cells/mm^2^), followed by AC with high-grade epithelial dysplasia (4.8 cells/mm^2^). Statistically significant differences were found in the density of intact mast cells compared to degranulated mast cells in AC with low-grade epithelial dysplasia (*p*<0.001), AC with high-grade epithelial dysplasia (*p*=0.005), and LLSCC (*p*<0.001). A positive correlation between degranulated mast cells and total inflammatory infiltrate (*p*=0.03) was observed in the LLSCC group. The expression of factor XIIIa+ dendrocytes was highest in AC with low-grade epithelial dysplasia (16.5 cells/mm^2^). The link between mast cell density, factor XIIIa+ dendrocytes, and inflammatory infiltrate indicates a potential crosstalk in lip carcinogenesis.

## Introduction

Lip cancer accounts for about 10% of all head and neck cancers and 0.2% of all cancers worldwide.^1^ Most lower lip squamous cell carcinomas (LLSCC) develop from actinic cheilitis (AC), which is recognized as an oral potentially malignant disorder.^2^ AC and LLSCC predominantly affect middle-aged and older adults with fair skin.^2,3^ AC is characterized by inflammation, scaling, and dryness of the lower lip and is linked to chronic exposure to ultraviolet (UV) radiation.^2,3^ UV radiation is responsible for inducing changes in the epithelium and connective tissue, leading to various degrees of epithelial dysplasia, solar elastosis, and inflammation, which can progress to LLSCC.^3,4^

Prior research has shown that UV radiation triggers the synthesis and release of mediators by mast cells, thereby influencing the degranulation process.^5,6^ Mast cell activation depends on the degranulation process initiated either by the IgE receptor or through interaction with various agonists that directly affect the cell surface, such as cytokines derived from T-cells.^7^ There is a strong correlation between mast cell density and both LLSCC and skin melanoma, hinting at UV-induced carcinogenesis via mast cell activation.^8–10^ Moreover, mast cell count is increased in AC compared to normal oral mucosa, suggesting a role for mast cells in the etiopathogenesis of AC.^9^ However, a recent study revealed that while increased mast cell density was associated with the severity of epithelial dysplasia in AC, it was not linked to areas of elastosis or collagen loss.^6^

Mast cell degranulation can impact the activity of dermal dendrocytes, including those expressing factor XIIIa, by releasing various bioactive molecules.^11,12^ The induction of factor XIIIa+ dendrocytes by mast cell degranulation has been observed in certain skin diseases such as psoriasis,^13^ acute urticaria,^12^ and Kaposi sarcoma.^14^ Dendrocytes are present in the oral mucosa and a subset of these dendrocytes near blood vessels express the protransglutamine-clotting enzyme factor XIIIa.^15,16^ Factor XIIIa+ dendrocytes have been detected in reactive and neoplastic oral lesions. The number, size, shape, and distribution of cells in perivascular areas in those lesions are clearly associated with inflammatory and hemostasis mechanisms in vessels.^15,16^ However, the interaction between mast cells and the expression of factor XIIIa+ dendrocytes in the pathogenesis of AC and the development of LLSCC remains unexplored.

The purpose of the present study was to evaluate mast cells and factor XIIIa+ dendrocytes to determine the density of these cells, as well as the relationship among mast cell degranulation, factor XIIIa expression by dendrocytes, and the inflammatory infiltrate in AC and LLSCC in comparison with normal oral mucosa.

## Methodology

### Study design and ethical clearance

This was a retrospective and cross-sectional study based on medical records from two Brazilian services of Oral and Maxillofacial Pathology: Universidade Federal de Pelotas (Pelotas, Rio Grande do Sul) and Universidade Federal de Minas Gerais (Belo Horizonte, Minas Gerais). The report of this study conformed to the Strengthening the Reporting of Observational Studies in Epidemiology (STROBE) statement.^17^ The study was approved by the Ethics Committee (No. 1328069) and the patients’ identities remained anonymous in accordance with the Declaration of Helsinki.

### Sample and diagnostic rendering

A total of 40 cases of AC, 50 of LLSCC, and 10 of normal oral mucosa (obtained from the retromolar region of patients undergoing third molar extraction and used as controls) were selected as a convenience sample. The inclusion criteria were LLSCC cases obtained from surgical treatment and AC cases diagnosed histopathologically as solar elastosis associated with low or high degrees of epithelial dysplasia.^18^ Five-μm-thick sections of formalin-fixed paraffin-embedded material obtained from each case were stained with hematoxylin and eosin (H&E) and independently reviewed by two oral and maxillofacial pathologists (R.A.M. and I.L.F.). Disagreements were resolved through consensus.

The exclusion criteria included records of patients with missing demographic information. Data regarding sex, age, skin color, anatomical location, clinical aspects of the lesions, lesion size, and evolution time were collected. Information on outdoor occupations was also collected, as some occupations typically involve long hours under direct sunlight, contributing to a higher risk of developing AC and LLSCC due to increased UV radiation exposure.

### Toluidine blue staining

The staining method for sulfated proteoglycans in secretion granules was performed to analyze mast cell metachromasia. Three-μm-thick sections were deparaffinized, rehydrated, and immersed in 0.1% toluidine blue (Sigma, St Louis, MO, USA) in 1% NaCl for 3 minutes. The slides were then washed in distilled water, dehydrated, and mounted. Mast cells were labeled according to the intensity of metachromasia and/or granule extrusion, as follows: an intact state was considered when there was a pronounced metachromasia without an evident nucleus, and/or without extrusion of granules around the cell. A degranulated state was considered when there was reduced metachromasia, an evident nuclear slope, and/or the presence of granules near the cell membrane.^19^

### Immunohistochemistry of factor XIIIa dendrocytes

Three-μm-thick sections were obtained from paraffin-embedded tissue blocks, mounted on polarized slides (StarFrost^®^, Waldemar Knittel Glasbearbeitungs GmbH, Germany), and subjected to immunohistochemistry. Analyses were performed using a monoclonal anti-factor XIIIa antibody (clone AC-1A1, IgG1; Abcam, Cambridge, UK; 1:50). The antigen-retrieval step was performed using citric acid at 95°C for 30 minutes in steamer. Endogenous peroxidase activity was blocked with hydrogen peroxide block (Spring Reveal kit; SPD-125 - Reveal - Biotin-Free Polyvalent DAB; Pleasanton, CA, USA) at 4°C for 16 hours. The reactions were developed with 3,3′-diaminobenzidine (DAB; Dako, Carpinteria, CA, USA), 0.1% dimethyl sulfoxide (DMSO), and 0.1% H_2_O_2_ for 2 minutes. Negative controls were conducted by omitting the primary antibody, thereby demonstrating the absence of labeling.

### Assessment of mast cells, factor XIIIa+ dendrocytes, and inflammatory infiltrate

All slides were scanned using a digital slide scanner system (3DHISTECH^®^, Budapest, Hungary) and further analyzed using the Pannoramic Viewer software (version 1.15.4; 3DHISTECH^®^, Budapest, Hungary). The assessments were conducted by a pre-trained examiner (I.L.F.) in a blinded fashion, with two separate evaluations performed two weeks apart. Intra-examiner analysis was conducted using the intraclass correlation coefficient test,^20^ which yielded a coefficient of 0.98.

Mast cells were counted at 40× magnification and mast cell density, i.e. intact cells/mm² and degranulated cells/mm², was calculated. The score was calculated in an area of 1 mm^2^ for cell count outlined manually in the subepithelial area in all samples.^19^ Factor XIIIa+ dendrocytes were counted as for mast cells, and 40× magnification was used to assess the positivity for factor XIIIa in dendrocytes. Factor XIIIa+ dendrocytes were counted in the subepithelial area as cells with brown staining of the cytoplasm with evident nuclei, and their density (positive cell number/mm²) was calculated.^21^ The inflammatory infiltrate (cell number/mm²) of neutrophils, lymphocytes, or plasma cells was calculated in H&E sections in an area of 1 mm^2^ for cell count outlined manually in the subepithelial area in all samples at 40× magnification.^21^

### Statistical analysis

The Statistical Package for the Social Sciences (SPSS) software (IBM SPSS Statistics for Windows, version 25.0, Armonk, NY: IBM Corp.) was used for statistical analysis of the data. The Kolmogorov-Smirnov test was used to analyze quantitative data distribution and the Mann-Whitney test was used to compare the density of intact and degranulated mast cells in the groups. The Kruskal-Wallis test was used to compare the density of intact and degranulated mast cells and the expression of factor XIIIa+ dendrocytes among groups. Spearman's rank correlation coefficients were calculated to determine the strength of the correlations of mast cells (intact, degranulated, and total), the expression of factor XIIIa+ dendrocytes, and the inflammatory infiltrate (neutrophils, lymphocytes, plasma cells, lymphoplasmacytic, and total) among all groups. The level of significance was set at 95% in all analyses.

## Results

### Clinicodemographic profile

The clinicodemographic data of the 100 patients are shown in Table 1. The survey predominantly included men (AC: n = 34/85% and LSCC: n = 48/96%). The mean age of AC patients with low- and high-grade epithelial dysplasia was 53 and 57.5 years, respectively, while the mean age of LLSCC patients was 63.5 years. All patients had fair skin (100%). The majority of patients with AC (n = 37/92.5%) and LLSCC (n = 45/90%) reported an outdoor occupation (*e.g.*, farmers, agricultural workers, construction workers, fishermen, maritime workers, gardeners). The lower lip was affected in all individuals with AC (n = 40/100%) and LLSCC (n = 50/100%). Clinically, all individuals with AC lesions presented with plaques/erosions (n = 40/100%), while the majority of individuals with LLSCC exhibited crusted ulcers (n = 39/78%). Most patients with AC (n = 28/70%) had lesions ≤ 2 cm in size, whereas those with LLSCC (n = 38/76%) had lesions between 2 and 4 cm. Regarding the time of evolution of LLSCC lesions, 31 (62%) individuals reported a duration of more than one year. In contrast, information about time of evolution of AC lesions was available in only three cases (7.5%). The control group (n = 10) was predominantly male (90%), white (100%), with a mean age of 21 years, and no outdoor occupation (100%).

**Table 1:** Clinical data of individuals with actinic cheilitis, lower lip squamous cell carcinoma, and normal oral mucosa.

Variables		Actinic cheilitis n = 40 (%)		Lower lip squamous cell carcinoma n = 50 (%)	Normal oral mucosa n =10 (%)
		Low-grade dysplasia n = 20	High-grade dysplasia n = 20		
Sex					
	Male	16 (80)	18 (90)	48 (96)	9 (90)
	Female	4 (20)	2 (10)	2 (4)	1 (10)
Age (mean ± SD)		53 ± 8.9	57.5 ± 11.6	63.5 ± 10.0	21 ± 3.7
Skin color					
	White	20 (100)	20 (100)	50 (100)	10 (100)
Outdoor occupation					
	Yes	18 (90)	19 (95)	45 (90)	–
	No	2 (10)	1 (5)	5 (10)	10 (100)
Clinical aspect					
	Plaque/erosion	20 (100)	20 (100)	–	–
	Crusted ulcer	–	–	39 (78)	–
	Ulcerative nodule	–	–	11 (22)	–
Size (cm)					
	≤ 2	17 (85)	11 (55)	8 (16)	–
	2–4	3 (15)	9 (45)	38 (76)	–
	> 4	–	–	4 (8)	–
Evolution time (year)					
	≤ 1	–	–	6 (12)	–
	> 1	–	3 (15)	31 (62)	–
	NR	20 (100)	17 (85)	13 (26)	–

NR: not reported; SD: standard deviation.

### Mast cell density was higher in LLSCC lesions than in AC patients and controls

**Figure 1:**
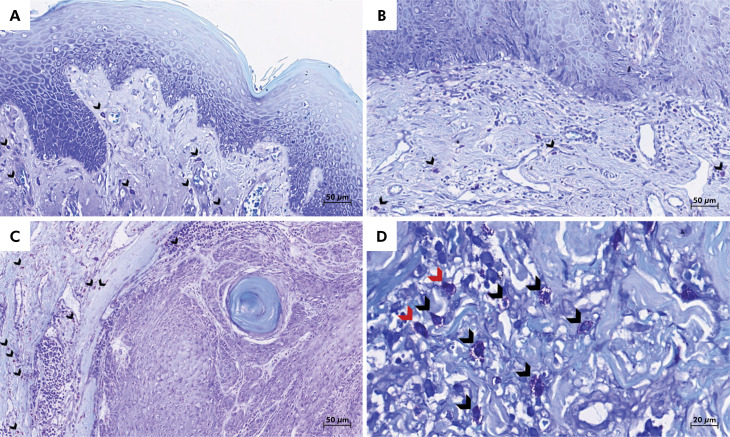
Representative images of toluidine blue-stained sections revealing mast cells (arrowheads) in (A) actinic cheilitis with low-grade epithelial dysplasia and (B) high-grade epithelial dysplasia, and (C) lower lip squamous cell carcinoma. (D) High-power view revealing degranulated (red arrowheads) and intact (black arrowheads) mast cells (toluidine blue; original magnification: ×40 and ×100).

While the median density of intact mast cells for LLSCC was 79 cells/mm^2^, AC with low-grade and high-grade epithelial dysplasia had a median of 69.5 and 55 cells/mm^2^, respectively. The median density of intact mast cells in normal oral mucosa was 35.5 cells/mm^2^. The median density of degranulated cells for LLSCC was 14 cells/mm^2^, 11 cells/mm^2^ for AC with high-grade and 9.5 cells/mm^2^ for low-grade epithelial dysplasia. The median density of degranulated mast cells in normal oral mucosa was 15 cells/mm^2^. Figure 1 depicts toluidine blue staining of AC with low- and high-grade epithelial dysplasia and LLSCC.

The median total mast cell density was 86 and 64 cells/mm^2^ in AC with low- and high-grade epithelial dysplasia, respectively, and 86 cells/mm^2^ for LLSCC. For normal oral mucosa, the median total mast cell density was 50.5 cells/mm^2^. The median density ratio of intact/degranulated mast cells was 3.5 and 4.8 cells/mm^2^ for AC with low- and high-grade epithelial dysplasia, respectively, and 5.9 cells/mm^2^ for LLSCC. For normal oral mucosa, the median density ratio of intact/degranulated mast cells was 2.1 cells/mm^2^ (Table 2). An association was found between intact mast cell density and degranulated mast cells density in all groups, including normal oral mucosa (p = 0.011), AC with low-grade epithelial dysplasia (p < 0.001), AC with high-grade epithelial dysplasia (p = 0.005), and LLSCC (p < 0.001). Similarly, a statistically significant difference was observed between intact mast cells and degranulated mast cells when considering the external cross-tabulation between normal oral mucosa, AC with low- and high-grade epithelial dysplasia, and LLSCC (Table 3).

**Table 2:** Density of intact, degranulated and total mast cells and expression of factor XIIIa+ dendrocytes in the actinic cheilitis, lower lip squamous cell carcinoma, and control.

Variables	Actinic cheilitis		Lower lip squamous cell carcinoma	Normal oral mucosa
	Low-grade dysplasia	High-grade dysplasia		
Mast cells (median and range; cells/mm^2^)				
Intact	69.5 (12–189)	55 (6–450)	79 (3–396)	35.5 (16–82)
Degranulated	9.5 (0–68)	11 (0–113)	14 (1–134)	15 (3–36)
Total	86 (19–201)	64 (16–522)	86 (6–463)	50.5 (37–85)
Ratio (intact/degranulated)	3.5 (0–27.3)	4.8 (0–55)	5.9 (0.2–85)	2.1 (0.4–27.3)
Factor XIIIa+ dendrocytes (median and range; cells/mm^2^)	16.5 (0–112)	9 (0–80)	15.5 (0–73)	13 (0–143)

**Table 3:** Association between density of intact and degranulated mast cells (median) in actinic cheilitis (AC) with low- and high-grade epithelial dysplasia, lower lip squamous cell carcinoma, and control.

Mast cells	Groups	p-value			
		AC with low-grade dysplasia (69.5 cells/mm^2^)	AC with high-grade dysplasia (55 cells/mm^2^)	Lower lip squamous cell carcinoma (79 cells/mm^2^)	Normal oral mucosa (35.5 cells/mm^2^)
Degranulated	AC with low-grade epithelial dysplasia (9.5 cells/mm^2^)	< 0.001	< 0.001	< 0.001	< 0.001
	AC with high-grade epithelial dysplasia (11 cells/mm^2^)	< 0.001	< 0.001	< 0.001	0.001
	Lip squamous cell carcinoma (14 cells/mm^2^)	< 0.001	< 0.001	0.640	0.001
	Normal oral mucosa (15 cells/mm^2^)	< 0.001	0.001	< 0.001	0.002

Spearman's rank correlation coefficient, p < 0.05.

### Expression of factor XIIIa+ dendrocytes in AC, LLSCC, and controls

**Figure 2:**
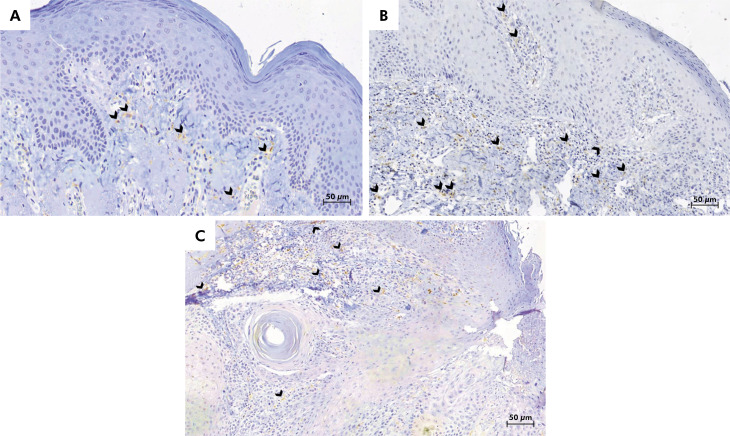
Representative immunohistochemical images of factor XIIIa+ dendrocytes-stained sections of (A) actinic cheilitis with low-grade epithelial dysplasia, (B) actinic cheilitis with high-grade epithelial dysplasia, and (C) lower lip squamous cell carcinoma. The cytoplasmic dendritic presentation of factor XIIIa+ dendrocytes can be seen (arrowhead) (3,3’-diaminobenzidine; original magnification: ×40).

The median density of factor XIIIa+ dendrocytes was highest in AC with low-grade epithelial dysplasia (16.5 cells/mm^2^), followed by LLSCC lesions (15.5 cells/mm^2^), and AC with high-grade epithelial dysplasia (9 cells/mm^2^). In normal oral mucosa, the median density of factor XIIIa+ dendrocytes was 13 cells/mm^2^ (Table 2). However, there was no statistically significant difference among groups in terms of factor XIIIa+ dendrocytes (p > 0.05). Figure 2 shows the cytoplasmic dendritic presentation of factor XIIIa+ dendrocytes in AC with low- and high-grade epithelial dysplasia and LLSCC.

### Correlation between density of degranulated mast cells and factor XIIIa+ dendrocytes and inflammatory infiltrate

A weak positive correlation between degranulated mast cells and factor XIIIa+ dendrocytes/mast cells was observed in the LSCCC group (r = 0.008; p = 0.979). In contrast, a moderate positive correlation was detected between degranulated mast cells and total inflammatory infiltrate (neutrophils, lymphocytes, and plasma cells) in the LLSCC group (r = 0.561; p = 0.03).

## Discussion

This study revealed a positive link between mast cell degranulation and factor XIIIa+ dendrocytes, contributing to the potentially malignant progression of AC to LLSCC. Mast cells were present in both AC and LLSCC, and the number of intact mast cells was higher in lesions than in normal oral mucosa. A correlation was found between degranulated mast cells and total inflammatory infiltrate and lymphoplasmacytic inflammatory infiltrate in LLSCC. Furthermore, an inflammatory component with mast cells was observed in the underlying stroma of AC, which may have contributed to the tenacity of the potentially malignant disorder, in addition to the progression and indolent course of LLSCC.

In 2022, 389,485 cases of lip and oral cavity cancer and 188,230 related deaths were reported worldwide.^22^ In Brazil, projections suggest approximately 15,100 new cases of oral cavity cancer annually from 2023 to 2025, translating to a risk of 6.99 per 100,000 inhabitants, with 10,900 cases in men and 4,200 in women.^23^ These figures underscore the severity of the situation, particularly in the Southeast and South of Brazil, regions that rank first in the frequency of lip and oral cavity cancer cases.^23^ The cases of AC and LLSCC examined in this study were from Minas Gerais and Rio Grande do Sul, states that have a high burden of lip and oral cavity cancer.^3,23,24^ These regions are characterized by tropical and subtropical climates, respectively, and provide a favorable environment for such conditions.^3,24^ For instance, a study conducted in Pelotas, a city from which a significant portion of our cases originate, revealed that 39.8% of men had sun exposure at work.^25^ This finding aligns with the observed trend of approximately 70% of AC and LLSCC cases occurring in men, with the lower lip being affected in 90% of cases.^3^ Moreover, sun exposure at work was more common among lower-income individuals in Pelotas and surrounding area,^25^ further emphasizing the link between outdoor occupations and increased risk of developing AC and LLSCC due to increased UV radiation exposure.^1–4^

Previous studies have revealed increased mast cell density in oral diseases such as lichenoid reaction and lichen planus.^15,16,26^ Additionally, the role of dendrocytes in immune surveillance in oral squamous cell carcinoma (OSCC) has been highlighted.^27^ Mast cell infiltration plays an important role in maintaining a chronic inflammatory infiltrate, particularly affecting tumor growth, invasion, and metastasis.^27,28^ Conversely, increased vascularity in OSCC suggests that angiogenesis begins in parallel with malignant transformation, which appears to be inversely linked to the number of mast cells.^27^ Mast cells also contribute to the maturation of potent antigen-presenting cells during interaction with factor XIIIa+ dendrocytes.^28^ Based on this, the hypothesis of a connection between mast cells and factor XIIIa+ dendrocytes in AC and LLSCC has been demonstrated in this study. This confirmation may be attributed to the observed positive association between mast cell degranulation and factor XIIIa+ dendrocyte activation. These findings support the notion that mast cells and factor XIIIa+ dendrocytes involved in the wound healing process are potential targets in the treatment of chronic diseases and cancer.^28^

In the present study, the toluidine blue technique was used to identify mast cells because of its effectiveness in providing a striking visual contrast, permitting a clear observation of a dense purple color in intact cells or a granulated pattern in degranulated cells.^29^ This analysis allowed us to identify a variation in density between intact and degranulated mast cells in all groups studied. Such findings, however, suggest an immune response both in the dysplastic epithelial mucosa and in the neoplastic process. Cell degranulation was lower in dysplastic events of the oral epithelium and in overt malignant lesions. Our results partially contrast with earlier data suggesting that degranulated mast cells may actively play a role in the progression of AC to LLSCC.^30^ However, the limited number of cases analyzed and the fact that the study was not a prospective cohort undermine the assessment of the relationship between density of degranulated mast cells and malignant transformation of AC into LLSCC. Interestingly, a recent study has documented that mast cell density increased significantly in AC cases with a high risk of severe epithelial dysplasia.^6^ The higher number of intact mast cells, as indicated by the intact/degranulated ratio, should be emphasized. This is noteworthy because, even though mast cells typically inhabit the dermis, their numbers can increase in response to inflammatory stimuli such as UV radiation. In LLSCC, the process of mast cell degranulation is closely associated with a chronic inflammatory infiltrate, which further supports the understanding of carcinogenic mechanisms.^5-7,10,21^ Nevertheless, potential differences between our results and those published elsewhere may be attributed to disparities in sample characteristics and, certainly, to the subjectivity inherent in the grading systems used to evaluate epithelial dysplasia.^6,18^

This study is apparently the first to explore the role of mast cell degranulation and its interaction with factor XIIIa+ dendrocytes in AC and LLSCC. This association has been previously described in conditions such as psoriasis and Kaposi sarcoma.^13,14^ In the latter, the hyperactivity of dendrocytes caused by the human immunodeficiency virus infection affects the local immune response rich in mast cells, contributing to its pathogenesis.^14^ In LLSCC samples, the correlation between degranulated mast cells and factor XIIIa expression, albeit weak, was positive. It is hypothesized that UV radiation evokes the activation mechanism between mast cells and factor XIIIa+ dendrocytes in LLSCC.^5^ However, the LLSCC profile may be associated with fluctuations in mast cell density, with a lower density of degranulated mast cells from dysplastic AC to LLSCC. A plausible explanation for this association is that the intensity and variability of the inflammatory infiltrate can influence mast cell behavior. This biological link has been formerly associated with immature and mature dendrocytes in LLSCC, supporting the idea of a stronger antitumor immunocytotoxic regulation in lip lesions compared to intraoral lesions. These findings are useful in explaining the lower occurrence of regional metastasis and a more favorable prognosis for these lesions.^31^ Further studies analyzing the set of these cellular components are encouraged, given the common inflammatory response in the tumor stroma of LLSCC.

Control samples were collected from the retromolar gingiva, which have the same type of keratinized stratified epithelium. Despite the lack of sun exposure in these samples, both intact and degranulated mast cells, as well as factor XIIIa+ dendrocytes, were detected. This finding aligns with another study that observed a higher density of total mast cells in gingival samples compared to other sites in the oral mucosa.^32^ These results suggest that mast cell activity, as indicated by degranulation, can be triggered by various stimuli, ranging from minor oral trauma to UV radiation exposure on the lower lip. Similarly, factor XIIIa+ dendrocytes have been identified in the lamina propria of gingival tissue.^33^ However, the predictive value of factor XIIIa+ dendrocytes and their association with mast cell degranulation and a high density of intact mast cells require further investigation using additional molecular techniques. These methods should aim to assess possible pathways involved in the microscopic stages of progression from AC to LLSCC.

The limitations of this study should be acknowledged. In some cases, the duration of lesion evolution was not reported, resulting in imprecise data on the potential progression from AC to LLSCC. This limitation is inherent to the retrospective design of the study. Additionally, data on the staging and grading of LLSCC were unavailable, as the study relied solely on diagnostic information from oral diagnostic services. After diagnosis, patients were referred to hospitals across the country, preventing the collection of data on staging, grading, or subsequent therapy. It is notable that recent literature indicates that patients diagnosed with oral and oropharyngeal cancer in Brazil typically take an average of 217 days to receive treatment.^34^ The greatest delays were attributed primarily to delays by healthcare professionals, followed by delays from patients in seeking medical assistance.^34^

## Conclusion

In summary, our findings reveal that progression to malignancy may be associated with slight fluctuations in the mast cell degranulation profile. The presence of inflammation may have triggered the increase in the intact mast cell population in AC and LLSCC. These results suggest that immune system cells show variable positivity in both epithelial dysplasia and neoplasia. The link between active mast cells through degranulation, factor XIIIa+ dendrocytes, and chronic inflammatory infiltrate reinforces the role of these immune cells in lip carcinogenesis.
